# Overexpression of KDM4 lysine demethylases disrupts the integrity of the DNA mismatch repair pathway

**DOI:** 10.1242/bio.201410991

**Published:** 2015-03-13

**Authors:** Samah W. Awwad, Nabieh Ayoub

**Affiliations:** Department of Biology, Technion – Israel Institute of Technology, Haifa 3200003, Israel

**Keywords:** DNA damage, KDM4 proteins, Mismatch repair, Chromosomal instability, Lysine demethylation

## Abstract

The KDM4 family of lysine demethylases consists of five members, KDM4A, -B and -C that demethylate H3K9me2/3 and H3K36me2/3 marks, while KDM4D and -E demethylate only H3K9me2/3. Recent studies implicated KDM4 proteins in regulating genomic instability and carcinogenesis. Here, we describe a previously unrecognized pathway by which hyperactivity of KDM4 demethylases promotes genomic instability. We show that overexpression of KDM4A-C, but not KDM4D, disrupts MSH6 foci formation during S phase by demethylating its binding site, H3K36me3. Consequently, we demonstrate that cells overexpressing KDM4 members are defective in DNA mismatch repair (MMR), as evident by the instability of four microsatellite markers and the remarkable increase in the spontaneous mutations frequency at the HPRT locus. Furthermore, we show that the defective MMR in cells overexpressing KDM4C is mainly due to the increase in its demethylase activity and can be mended by KDM4C downregulation. Altogether, our data suggest that cells overexpressing KDM4A-C are defective in DNA MMR and this may contribute to genomic instability and tumorigenesis.

## INTRODUCTION

A decade ago, two families of lysine demethylases (KDM) have been identified, confirming that lysine methylation is a reversible and dynamically regulated process (Klose et al., 2006a; Shi et al., 2004; Whetstine et al., 2006). One family is referred to as the Jumonji C (JmjC)-domain-containing proteins. The crystal structure of the JmjC catalytic domain was solved and found to form an enzymatically active pocket that coordinates the two main co-factors needed for the radical-based oxidative demethylation reaction, ferrous oxide (Fe(II)) and α-ketoglutarate (Chen et al., 2006; Shi and Whetstine, 2007; Tsukada et al., 2006).

The human KDM4A-E family (also known as JMJD2A-E) consists of five members, which specifically catalyze the demethylation of H3K9me2/me3. Furthermore, KDM4A, -B and -C, but not KDM4D and -E, demethylate H3K36me2/me3 and H1.4K26me2/me3 (Chen et al., 2006; Labbé et al., 2013; Trojer et al., 2009). KDM4A-E proteins are involved in multiple cellular processes including gene expression regulation (Kim et al., 2012; Mallette and Richard, 2012; Shin and Janknecht, 2007a; Wissmann et al., 2007; Zhang et al., 2005), DNA replication (Black et al., 2010; Black et al., 2012), and DNA damage response (Khoury-Haddad et al., 2014; Mallette et al., 2012; Palomera-Sanchez et al., 2010; Young et al., 2013; Black, 2012), worm development and germ cell apoptosis (Whetstine et al., 2006), renewal of embryonic stem cells (Loh et al., 2007), and male life span in drosophila (Lorbeck et al., 2010). Interestingly, increasing number of reports implicate KDM4 misregulation in promoting genomic instabilities and carcinogenesis (Berdel et al., 2012; Berry and Janknecht, 2013; Black et al., 2013; Cloos et al., 2006; Ehrbrecht et al., 2006; Italiano et al., 2006; Kawazu et al., 2011; Labbé et al., 2013; Li et al., 2011; Liu et al., 2009; Luo et al., 2012; Northcott et al., 2009; Shi et al., 2011; Vinatzer et al., 2008; Wissmann et al., 2007; Yang et al., 2000; Zack et al., 2013).

A recent report implicated H3K36me3 mark in DNA mismatch repair (MMR). It demonstrated that the mismatch recognition protein hMutSα binds H3K36me3 during early S phase to ensure intact DNA MMR (Li et al., 2013). These observations prompted us to investigate the role of KDM4 proteins in DNA MMR. Here, we describe a previously unrecognized pathway by which upregulation of KDM4 proteins promotes genomic instability. We show that overexpression of KDM4 impairs the integrity of DNA mismatch repair (MMR) and thus leading to microsatellite instability (MSI) and to an increase in the frequency of spontaneous mutations. Interestingly, we show that downregulation of KDM4C expression restores the integrity of DNA MMR. Collectively, our data provide a new pathway by which KDM4A-C amplification may lead to genomic instability and tumorigenesis.

## RESULTS AND DISCUSSION

### KDM4A-C overexpression disrupts MSH6 foci formation during S-phase

**Fig. 1.**
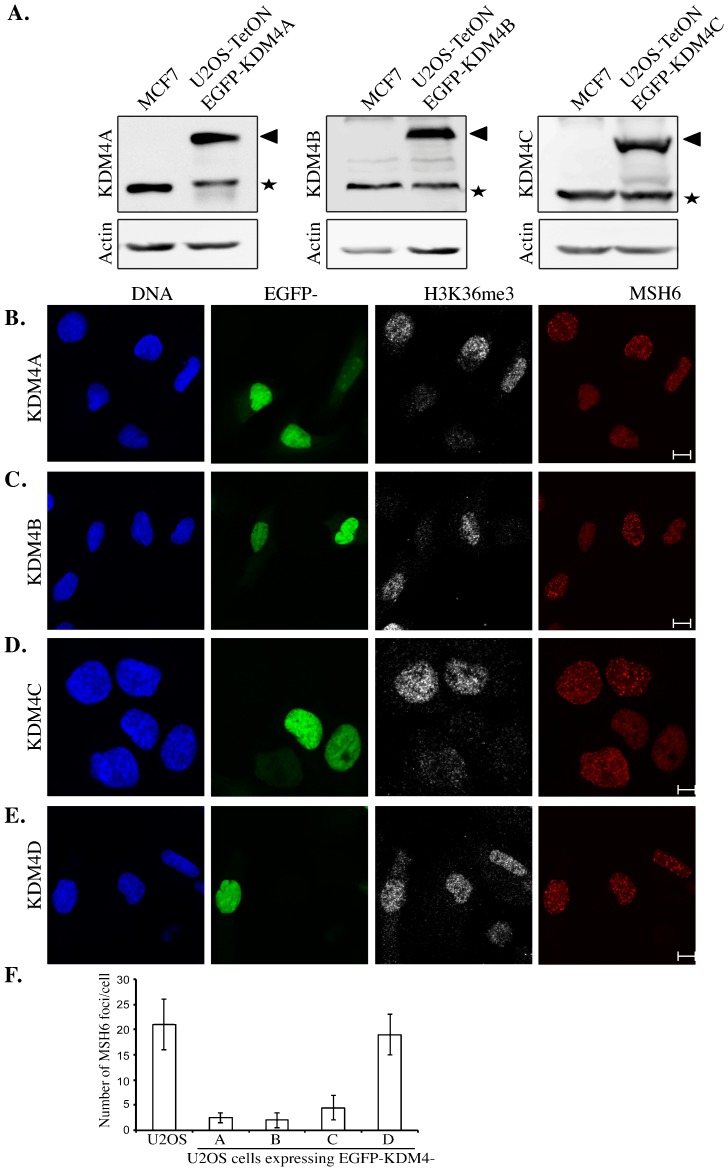
Overexpression of KDM4A-C, but not KDM4D, proteins impairs MSH6 foci formation during S-phase. (A) Western blot analysis shows the levels of EGFP-KDM4A-C fusions in U2OS-TetON cell lines in comparison to the levels of the endogenous KDM4A-C proteins in MCF7 cell line. Protein extracts were prepared from MCF7 cell line and from doxycycline-treated U2OS-TetON cells expressing EGFP-KDM4A-C fusions and immunoblotted using the indicated antibodies. β-actin is used as a loading control. EGFP-KDM4A-C fusions and the endogenous KDM4A-C proteins are indicated by arrowheads and stars, respectively. (B–E) Shows that overexpression of EGFP-KDM4A-C, but not EGFP-KDM4D, catalyzes the removal of H3K36me3 methylation and impairs MSH6 foci formation during S phase. Cells were fixed and subjected to immunofluorescence analysis using antibodies against MSH6 (red) and H3k36me3 (gray). DNA is stained with DAPI (blue) and EGFP-KDM4A-D fusions are in green. Results shown in (B–E) are typical of two independent experiments and represent at least 30 different cells each. (F) Graph shows the number of MSH6 foci in untransfected U2OS cells and in U2OS cells expressing EGFP-KDM4A-D fusions (n = 30 cells). Foci were counted by eye. Error bars represent SD from two independent experiments. Scale bars = 10 µm (B,C,E); 5 µm (D).

KDM4A-C proteins, but not KDM4D, demethylate H3K36me3 mark as we and others have shown (Couture et al., 2007; Hillringhaus et al., 2011; Klose et al., 2006b; Kupershmit et al., 2014; Shin and Janknecht, 2007b; Whetstine et al., 2006). H3K36me3 is involved in DNA MMR as it provides a binding site for the MMR protein MSH6 and enables MSH6 foci formation during S phase (Li et al., 2013). Therefore, we sought to assess whether overexpression of KDM4A-C proteins affects MSH6 foci during S phase. Toward this end, we used U2OS-TetON cell lines that conditionally express functional EGFP-KDM4A-C fusions upon the addition of doxycycline (Ipenberg et al., 2013; Kupershmit et al., 2014). Importantly, the expression levels of EGFP-KDM4A-C fusions are comparable to the levels of the endogenous KDM4A-C proteins found in human breast adenocarcinoma cell line, MCF7, known to have elevated levels of KDM4 proteins (Berry and Janknecht, 2013; Berry et al., 2012) (Fig. 1A). The cells were synchronized at G1/S border using double-thymidine block; samples were collected at 3 hr after the removal of thymidine and subjected to both fluorescence-activated cell sorter (FACS) and immunofluorescence (IF). Results show that 3 hr after thymidine removal the majority of the cells (83%) were at S phase (supplementary material Fig. S1). IF analysis shows that overexpression of EGFP-KDM4A-C fusions (green) diminished the intensity of H3K36me3 signal (gray) and impaired MSH6 foci during S phase (red) (Fig. 1B–D). On the other hand, U2OS-TetON cells expressing EGFP-KDM4D fusion (Khoury-Haddad et al., 2014), which does not demethylate H3K36me3, show no detectable effect on H3K36me3 levels and MSH6 foci (Fig. 1E). Quantitative measurements of the MSH6 foci reveal that KDM4A-C overexpression leads to 6–9 fold decrease comparing to control U2OS cell line or cells overexpressing KDM4D (Fig. 1F). We concluded therefore that EGFP-KDM4A-C overexpression leads to a dramatic reduction in H3K36me3 and impairs MSH6 foci during the S phase of the cell cycle. Our results are consistent with a recent report showing that MSH6 foci during S phase is impaired following the inhibition of H3K36me3 methylation by knocking down SETD2 methyltransferase (Li et al., 2013).

### Overexpression of KDM4 members impairs the integrity of DNA mismatch repair

**Fig. 2.**
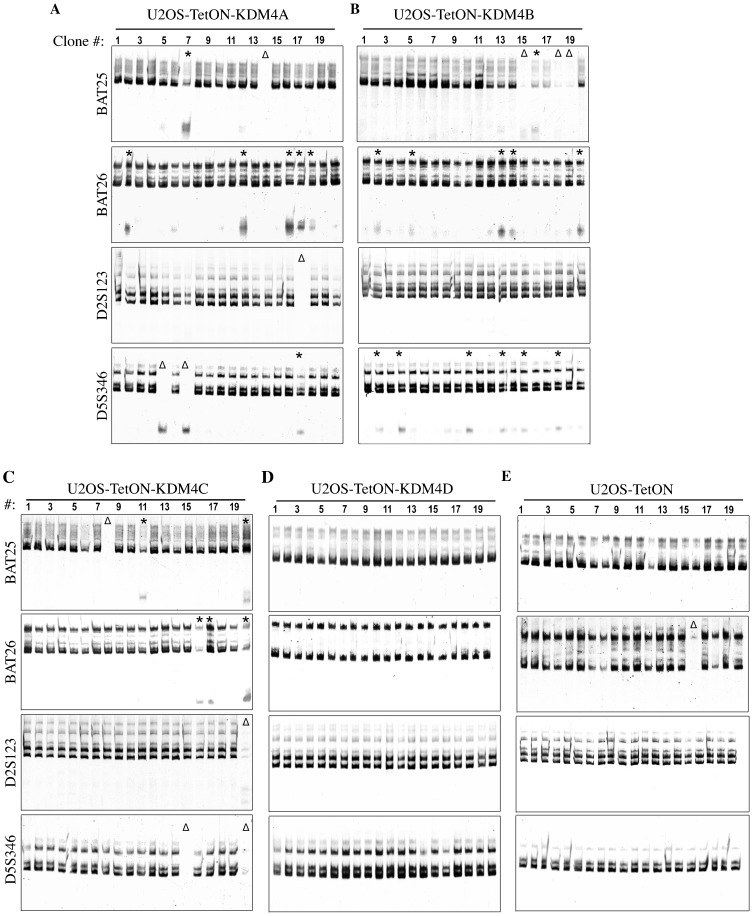
Overexpression of KDM4A-C, but not KDM4D, displays MSI phenotype. (A–E) Microsatellite instability (MSI) assay showing the analysis of PCR product patterns of four microsatellite markers in subclones derived from U2OS-TetON expressing EGFP fused to KDM4A (A), KDM4B (B), KDM4C (C), KDM4D (D) and U2OS-TetON control cells (E). To determine the microsatellite stability, genomic DNA was extracted from 20 single clones derived from different single cells of each cell line. The indicated microsatellite markers were then amplified using specific primers pairs, resolved by polyacrylamide-urea electrophoresis and visualized by SYBR-Gold staining. Δ and * show clones exhibiting complete deletion of the tested microsatellite markers or new repeat species, respectively.

It was shown that H3K36me3-MSH6 interaction is essential for intact DNA MMR (Li et al., 2013). We predicted therefore that the removal of H3K36me3 mark following KDM4A-C overexpression should disrupt the integrity of DNA MMR. Given that microsatellite instability (MSI) is a common hallmark of MMR-defective cells (Boland et al., 1998; Bocker et al., 1997; Ellegren, 2004; Hsieh and Yamane, 2008), we sought to test the stability of the mononucleotide (BAT25, BAT26) and the dinucleotide (D2S123, D5S346) microsatellite markers in U2OS-TetON cell lines overexpressing EGFP-KDM4A-D proteins. MSI assay was performed on genomic DNA, which was extracted from 20 clones derived from different single cells of each cell line. Results show that cells overexpressing KDM4A-C, but not KDM4D, exhibit MSI as evidence by the appearance of new repeat species (marked by *) and complete deletions of the tested markers (marked by Δ). As shown in Fig. 2A, 40% (8/20 clones), 55% (11/20) and 30% (6/20) of the clones expressing KDM4A, B and C respectively, show either deletion or novel microsatellite mark. On the other hand, no detectable alterations in the length of the four tested microsatellite markers were obtained in clones overexpressing KDM4D (Fig. 2D) and only one clone shows deletion in the control U2OS-TetON cells (Fig. 2E).

Clones with two or more abnormal microsatellite marker are scored as MSI-high (MSI-H), while MSI-low clones have only one unstable marker (Boland et al., 1998; Haydon and Jass, 2002; Merok et al., 2013). Based on this classification, we observed that U2OS-TetON-EGFP-KDM4A cells contain two MSI-H clones, clone #7 (shows instability in 2 out of 4 markers) and clone#17 (shows instability in 3 out of 4 markers) (Fig. 2A). Further, U2OS-TetON-EGFP-KDM4B cells contain 4 MSI-H clones, clone #2, clone#13, clone#15 and clone#18 (each shows instability in 2 out of 4 markers) (Fig. 2B). Further, clone#20 of U2OS-TetON-EGFP-KDM4C cells shows instability in all four tested markers (Fig. 2C). On the other hand, no MSI-H clones were observed in U2OS-TetON-EGFP-KDM4D (Fig. 2D) and in U2OS-TetON control cells (Fig. 2E).

Notably, the sizes of the observed new microsatellite alleles are similar (Fig. 2). This could be due to continuous overexpression of KDM4A-C protein for prolong time (2–3 weeks) which leads to selective growth advantage of clones with a certain length of microsatellite markers. To address this possibility, we repeated the MSI assay on cells that overexpressed EGFP-KDM4C for four days only. Results show that 85% of the clones are MSI-H. Interestingly, unlike the situation in Fig. 2C, we observed novel alleles with heterogeneous repeat length, suggesting that indeed prolong expression of KDM4C may lead to the appearance of novel alleles with uniform size (supplementary material Fig. S2). Altogether, our data suggest that overexpression of KDM4A-C, but not KDM4D, disrupts the integrity of DNA MMR and leads to MSI.

A second readout of a defective DNA MMR is the increase in the spontaneous mutation frequency (Albertini, 2001; Branch et al., 1995; Eshleman et al., 1995). Therefore we performed HPRT mutability assay to determine the mutation frequency at the HPRT locus in cells overexpressing KDM4A-D members (see Materials and Methods; supplementary material Fig. S3). Results show that KDM4A-C overexpression leads to a dramatic increase in the mutation frequency which is between ∼500–1000 folds comparing to the control U2OS-TetON cells (Table 1). Surprisingly, cells overexpressing KDM4D show also significant increase in the mutation frequency (32.5 fold increase), however it is at least 15 fold less than the increase observed in cells overexpressing KDM4A-C fusions (Table 1). Altogether, we concluded that overexpression of KDM4A-C proteins leads to a remarkable increase in the mutation frequency, highlighting the key role of H3K36me3 in regulating the DNA MMR pathway. In addition, the increased mutation frequency in cells overexpressing KDM4D suggests that methylation marks other than H3K36me3 might be also implicated in regulating the fidelity of DNA MMR. Interestingly, the proteins levels of the four key MMR genes MSH6, MSH2, MLH1 and PMS2 are not reduced in cells overexpressing KDM4 proteins (supplementary material Fig. S4), suggesting that the defective DNA MMR is not due to decrease in the levels of the tested key MMR proteins. Notably, overexpression of KDM4A-D proteins leads to 1.9 and 1.5 fold increase in the protein levels of MLH1 and MSH6, respectively.

**Table 1.**
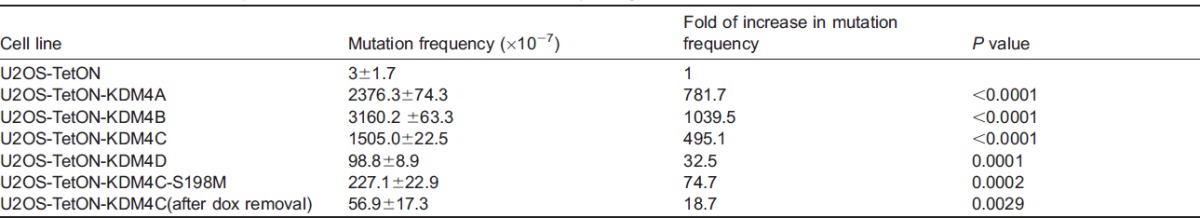
KDM4A-D overexpression increases the mutation frequency at the HPRT locus

### Overactivity of KDM4C demethylase disrupts the integrity of DNA MMR

**Fig. 3.**
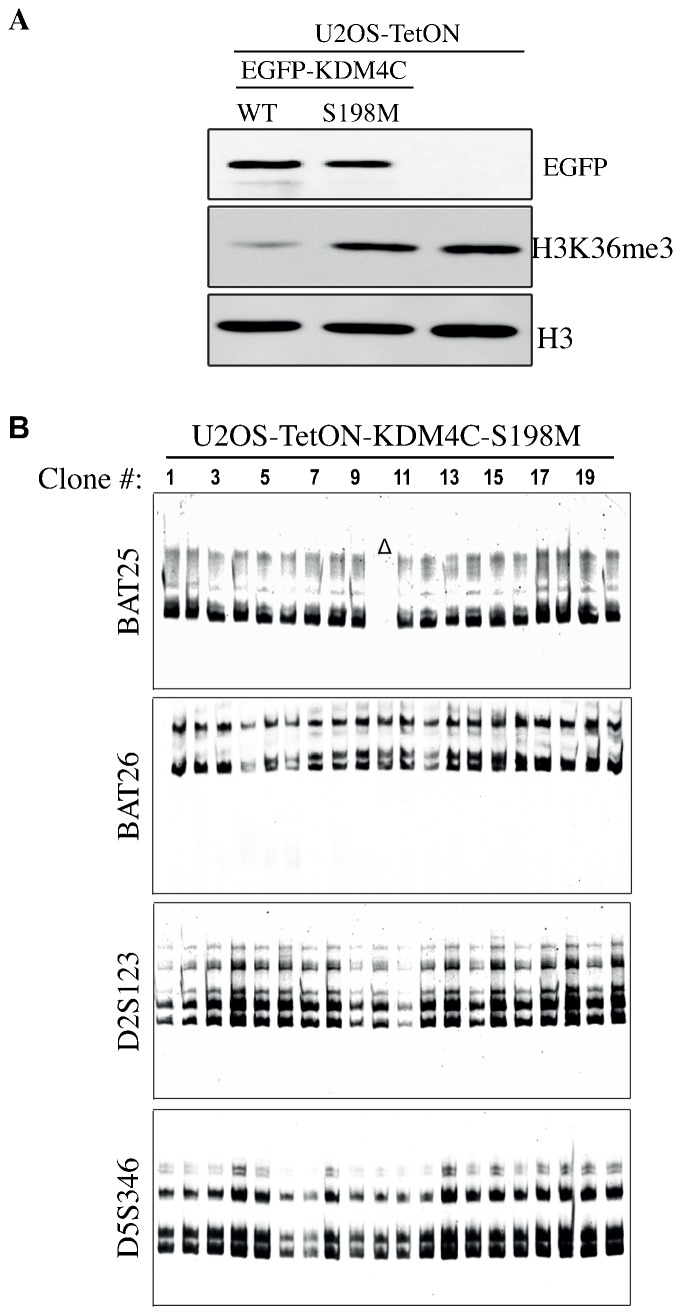
KDM4C over-activity disrupts DNA MMR. (A) Western blot analysis showing that overexpression of EGFP-KDM4C-S198M has no detectable effect on the levels of H3K36me3. Protein extracts were prepared from U2OS-TetON cells expressing either EGFP-KDM4C-WT or EGFP-KDM4C-S198M and immunoblotted using the indicated antibodies. (B) MSI assay was performed as described in Fig. 2 except that genomic DNA was extracted from 20 single clones derived from U2OS-TetON cells expressing KDM4C-S198M. Results show that only one clone out of 20 clones shows instability in a single marker. Δ shows clones exhibiting complete deletion of the tested microsatellite markers.

We show that the decrease in H3K36me3 levels following KDM4A-C overexpression impairs MSH6 foci formation and disrupts the integrity of DNA MMR (Figs 1, 2 and Table 1). These findings suggest that the levels of H3K36me3 have a critical role in regulating DNA MMR pathway. To further validate this, we monitored the integrity of MMR in cells overexpressing KDM4C demethylase-dead mutant. Previously, we have established a catalytically inert KDM4C-S198M mutant that does not demethylate H3K9me3 (Kupershmit et al., 2014). Here, we validated by western blot that KDM4C-S198M overexpression also has no effect on the levels of H3K36me3 (Fig. 3A). To assess the integrity of MMR, cells overexpressing KDM4C-S198M were subjected to MSI and HPRT mutability assays. The MSI results show no MSI-H clones and only one out of the 20 tested clones overexpressing KDM4C-S198M shows complete deletion of BAT25 marker (Fig. 3B). In addition, the HPRT mutability assay shows that the mutation frequency in cells overexpressing KDM4C-S198M is ∼7 fold less than in cells overexpressing KDM4C-WT (Table 1). Nonetheless, KDM4C-S198M overexpression shows significant increase in the mutation frequency (∼75 fold) at the HPRT locus comparing to U2OS-TetON cells. Altogether, our findings confirm that the defective DNA MMR in cells overexpressing KDM4 results mainly from the increase in the demethylase activity. However, overexpression of the catalytically inert mutant has also a relatively minor effect on the integrity of DNA MMR. This result suggests that one mechanism by which KDM4C-S198M interferes with MMR might be by competing with MSH6 on binding to H3K36me3.

### The KDM4C-dependent MMR defect is mended by KDM4C downregulation

**Fig. 4.**
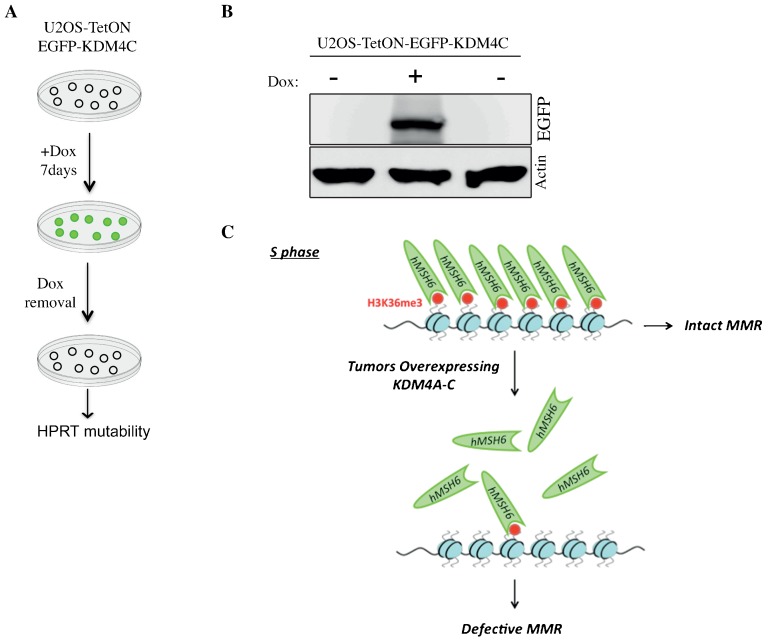
Downregulation of KDM4C restores the integrity of DNA mismatch repair. (A) An outline describing the experimental flow to assess the effect of KDM4C downregulation on the rate of mutation frequency at the HPRT gene. Cells were cultured with doxycycline for 7 days to induce EGFP-KDM4C expression, then the doxycycline was removed to shutdown the expression of EGFP-KDM4C and subsequently the mutation frequency at the HPRT gene was determined. (B) Western blot showing that doxycycline removal suppresses the expression of EGFP-KDM4C fusion. Protein lysates from untreated and doxycycline-treated U2OS-TetON-EGFP-KDM4C cells were immunoblotted using the indicated antibodies. (C) A model showing that KDM4A-C overexpression diminishes H3K36me3 signal, impairs MSH6 foci and impairs the integrity of DNA MMR pathway.

Here, we asked whether the defective MMR in cell overexpressing KDM4C could be repaired by KDM4C downregulation. To address this question, we treated cells with doxycycline for five days to trigger EGFP-KDM4C overexpression and disrupt DNA MMR. Next, EGFP-KDM4C expression was shutdown by doxycycline removal and cells were subjected to HPRT mutability assay (Fig. 4A). To validate the shutting down of EGFP-KDM4C expression, we performed western blot before and after doxycycline removal (Fig. 4B). The HPRT mutability showed that the removal of doxycycline leads to a decrease of ∼26 fold in the mutation frequency comparing to cells overexpressing KDM4C protein (Table 1). This result strongly suggests that KDM4C downregulation restores the integrity of DNA MMR. Given that Microsatellite-stable (MSS) and MSI tumors display distinct responses to certain antitumor agents (Merok et al., 2013; Ribic et al., 2003; Vilar et al., 2008; Vilar and Tabernero, 2013), we speculate that small molecule inhibitors of KDM4C may restore the integrity of DNA MMR and alter the sensitivity to anticancer drugs.

Here we reveal a previously unrecognized role of KDM4A-C upregulation in modulating the fidelity of DNA MMR pathway. The model that emerges from our results suggests that defective MMR in cells overexpressing KDM4A-C is primarily due to the elevation of their demethylase activity, which is accompanied by a dramatic decrease in the levels of H3K36me3 and loss of MSH6 foci formation during S phase (Fig. 4C). Our observations are consistent with previous report showing that depletion of SETD2 methyltransferase reduces H3K36me3 levels, impairs MSH6 foci formation and disrupts DNA MMR (Li et al., 2013).

Interestingly, while KDM4D overexpression has no detectable effect on the stability of the four tested microsatellite markers (Fig. 2D), it leads to 32.5 fold increase in the mutation frequency at the HPRT locus (Table 1). This increase is independent of H3K36me3-MSH6 interactions because H3K36me3 levels are not affected by KDM4D overexpression (Fig. 1E). The KDM4D-dependent increase in the mutation frequency might be related to: (i) changes in the methylation marks of either histone other than H3K36me3 and/or non-histone proteins. Indeed, it was recently shown that KDM4D demethylates H3K56me3 (Jack et al., 2013) and non-histone substrates (Ponnaluri et al., 2009; Yang et al., 2011). (ii) Changes in the expression patterns of KDM4D target genes that might be implicated in the DNA MMR pathway. Future studies will be required to further clarify this issue.

## MATERIALS AND METHODS

### Cell lines and growth conditions

All cell lines were grown in Dulbecco's modified Eagle's medium (DMEM) as previously described (Ipenberg et al., 2013; Khoury-Haddad et al., 2014; Kupershmit et al., 2014).

### Cell synchronization

U2OS-TetON-EGFP-KDM4A-D cells were synchronized at G1/S by double thymidine block as previously described (Lee et al., 2004).

### Flow cytometry

FACS analysis was performed as previously described (Khoury-Haddad et al., 2014). DNA content was analyzed using flow cytometry of 10,000 events on a BD LSR-II flow cytometer (Becton Dickinson), equipped with FCS Express software. Data were analyzed using ModFit LT 3.3 software.

### Western blotting

Western blotting was performed as previously described (Khoury-Haddad et al., 2014). Briefly, protein lysates were prepared using hot-lysis and immunoblotted using the appropriate antibodies (a complete list of antibodies and their dilutions used in this study are described in supplementary material Table S1). Membranes were developed using Quantum ECL detection kit (K-12042-D20, Advansta).

### Immunofluorescence

Cells were grown on coverslips and subjected to immunofluorescence as previously described (Khoury-Haddad et al., 2014). Cells were stained with the appropriate antibodies (supplementary material Table S1). Slides were visualized using the inverted Zeiss LSM 700 confocal microscope with 40× oil EC Plan Neofluar objective.

### Microsatellite instability assay (MSI)

U2OS-TetON control cell line and U2OS-TetON cell lines expressing EGFP-KDM4A-D and EGFP-KDM4C-S198M fusions were plated at limiting dilutions for an average density of 100–500 cells into 15 cm plates. By 2–3 weeks after plating, genomic DNA was extracted from 20 single clones of each cell line using the NucleoSpin Tissue XS kit. MSI assay was performed as previously described (Bocker et al., 1997; Boland et al., 1998). In brief, four microsatellite markers (BAT25, BAT26, D2S123 and D5S346) were amplified using PCR, the amplified PCR products were resolved by electrophoresis in an 8% polyacrylamide gel containing 7.7 M urea, stained with SYBR Gold (Life Technologies 1308457) and visualized using the Gel Doc™ XR^+^ imaging system.

### HPRT mutability assay

The HPRT mutability assay was performed as described in (Glaab et al., 1998; Glaab and Tindall, 1997; Kat et al., 1993), except for the following changes. Cells were cultured in growth medium containing 100 µM hypoxanthine, 0.4 µM aminopterin and 16 µM Thymidine (HAT) for 5 days to eliminate the pre-existing HPRT mutant cells. Next, the cells were transferred to HAT-free medium and treated with doxycycline for 3 days to trigger the expression of the EGFP-KDM4 fusions. Afterward, 9×10^6^ cells were plated in medium containing 5 µM 6-Thioguanine (6-TG; Sigma#A4660) and 1 µg/ml doxycycline at a density of 3×10^6^ per 10 cm dish. In addition, 1.5×10^3^ cells were plated at a density of 500 cells per 6 cm dish containing doxycycline but without 6-TG to determine plating efficiency. The plates were incubated at 37°C in humidified incubator for 21–30 days. The colonies were visualized by staining with 0.005% crystal violet and colonies containing more than ∼50 individual cells are counted using a stereomicroscope. The mutation frequency (MF) was calculated according to the following equation: MF = a/(9×10^6^×[b/1.5×10^3^]). a = total number of 6-TG resistant colonies and b = total number of colonies observed in the plating efficiency plates.

## Supplementary Material

Supplementary Material
