# Supplementary material for: Overexpression of KDM4 lysine demethylases disrupts the integrity of the DNA mismatch repair pathway
Source: Biol Open. 2015 Mar 13;4(4):498–504. doi: 10.1242/bio.201410991 (PMC4400592; doi:10.1242/bio.201410991)
Supplement: Supplementary Material [file supp_4_4_498__index.html]

Overexpression of KDM4 lysine demethylases disrupts the integrity of the DNA mismatch repair pathway — Overexpression of KDM4 lysine demethylases disrupts the integrity of the DNA mismatch repair pathway — Supplementary Material 

# Overexpression of KDM4 lysine demethylases disrupts the integrity of the DNA mismatch repair pathway

## bio.201410991 Supplementary Material

**Files in this Data Supplement:**

- Supplementary Material - Samah W. Awwad and Nabieh Ayoub doi: 10.1242/bio.201410991
