# Supplementary material for: Overexpression of KDM4 lysine demethylases disrupts the integrity of the DNA mismatch repair pathway
Source: Biol Open. 2015 Mar 13;4(4):498–504. doi: 10.1242/bio.201410991 (PMC4400592; doi:10.1242/bio.201410991)
Supplement: Supplementary Material [file supp_bio.201410991_bio.201410991-s1.pdf]

Biology Open (2015) 000, 1–7 doi:10.1242/bio.201410991

Supplementary Material

Samah W. Awwad and Nabieh Ayoub doi: 10.1242/bio.201410991

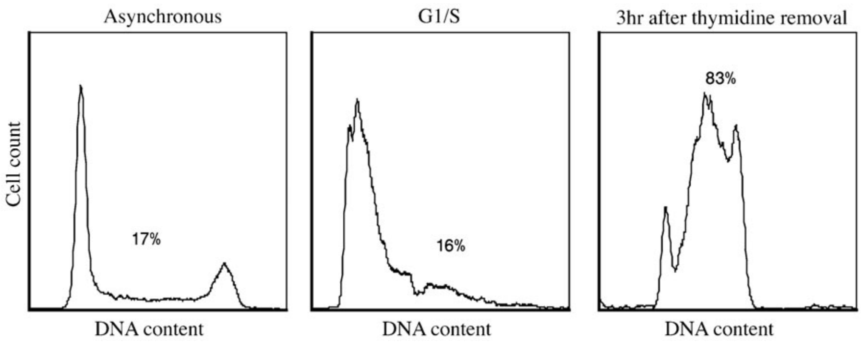

Fig. S1. Cell synchronization using double-thymidine block. Flow cytometric analysis shows the enrichment of cells at S phase at 3 hours after thymidine removal. U2OS-TetON cells expressing EGFP-KDM4A fusion were synchronized at G1/S border using double-thymidine block. To allow entry of the cells into S phase, thymidine was removed and samples were collected at three hours and stained with propidium iodide to monitor cell cycle distribution using fluorescence-activated cell sorter (FACS). Results show that 83% of the cells are in the S phase.

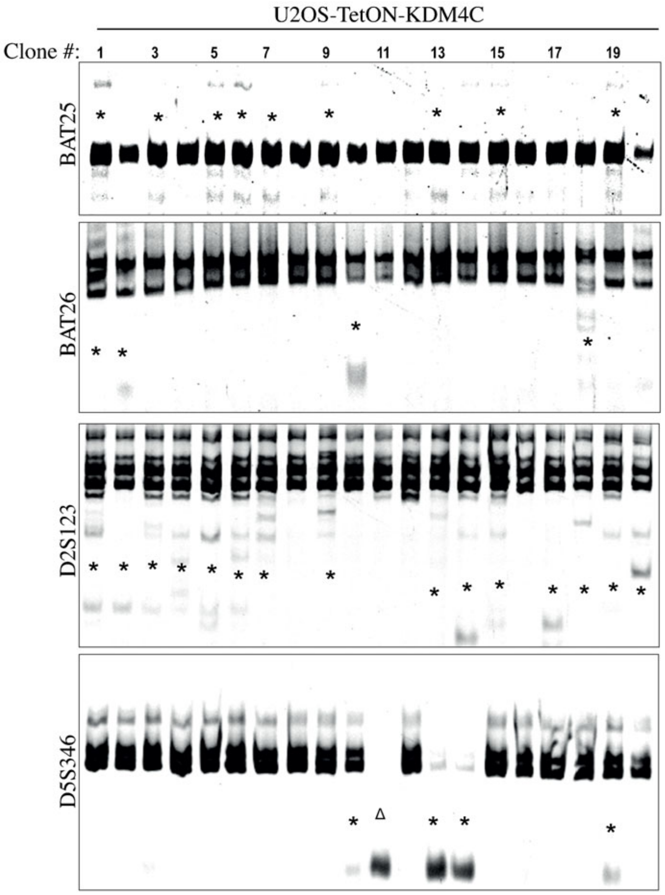

Fig. S2. Overexpression of KDM4C displays MSI phenotype. MSI assay was performed as described in Fig. 2 except that the cells were treated with doxycycline for four days (instead of 3 weeks) to induce the expression of EGFP-KDM4C fusion. Δ and * show clones exhibiting complete deletion of the tested microsatellite markers or new repeat species, respectively.

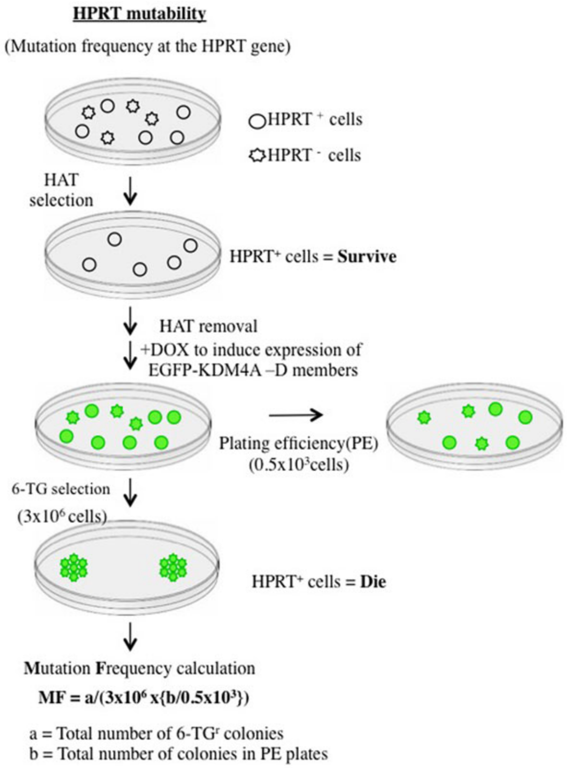

Fig. S3. Experimental flow of the HPRT mutability assay. U2OS-TetON and U2OS-TetON-EGFP-KDM4A-D cells were cultured in the presence of HAT containing medium for 5 days to eliminate the HPRT mutant cells. Next, the remaining HPRT wild-type cells were treated with doxycycline to induce the expression of EGFP-KDM4A-D fusions and 3 million cells were plated in 10 cm dish in the presence of doxcycline and 5 µM 6-TG and for 2–3 weeks to select for HPRT mutant cells. In parallel, 500 cells were plated in the absence of 6-TG to determine the plating efficiency. Mutation frequency was then determined by dividing the number of 6-TG resistant clones by the total number of plated cells as described in Materials and Methods.

Biology Open (2015) 000, 1–7 doi:10.1242/bio.201410991

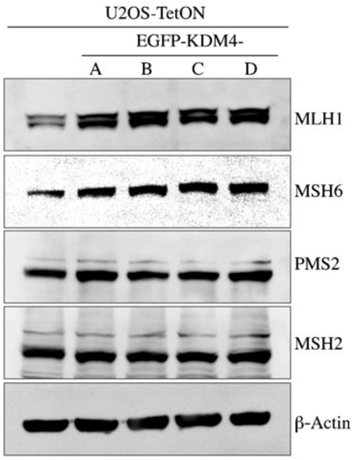

Fig. S4. The proteins levels of the key MMR genes are not reduced following KDM4A-D overexpression. Western blot analysis shows the protein level of MSH6, MSH2, PMS2 and MLH1 in cells overexpressing KDM4A-D proteins and in control U2OS-TetON cells. Protein lysates were prepared using hot-lysis, resolved and immunoblotted using the indicated antibodies and Anti-β-actin as a loading control. The bands intensities of MSH6, MSH2, PMS2 and MLH1 were normalized relative to the intensities of their respective β-actin bands and the ratios are shown at the bottom of the blot.

Table S1. List of antibodies used in this study

Name	Source	Dilution for western blot	Dilution for immunofluorescence
Primary antibodies			
Anti-H3	abcam #ab1791	1:2000-20,000	
Anti-β-Actin	SIGMA #A5441	1:15000	
Anti-GFP	Abcam #ab290	1:2000	
Anti-H3K36me3	Abcam #ab9050	1:3000	1:1000
Anti-MSH6	Cell Signaling #12988	1:500	1:500
Anti-KDM4A	In-house	1:400	
Anti-KDM4B	In-house	1:800	
Anti-MSH2	Santa Cruz #494	1:500	
Anti-PMS2	Santa Cruz #618	1:500	
Anti-MLH1	Santa Cruz #582	1:500	
Anti-KDM4C	Novus-NBP1-49600	1:1000	
Secondary antibodies			
Anti-mouse(IgG)-HRP	Amersham #NAX931	1:10,000	
Anti-rabbit(IgG)-HRP	Jackson ImmunoResearch #111-035-003	1:20,000	
Donkey anti-mouse-Alexa Flour®488	Invitrogen #A21202		1:500
Donkey anti-rabbit DyLightTM649	Jackson ImmunoResearch #711-495-152		1:500
